# German-speaking medical students on international electives: an analysis of popular elective destinations and disciplines

**DOI:** 10.1186/s12992-021-00742-z

**Published:** 2021-08-16

**Authors:** Maximilian Andreas Storz, Ann-Kathrin Lederer, Eric Pieter Heymann

**Affiliations:** 1 Center for Complementary Medicine, Institute for Infection Prevention and Hospital Epidemiology, Medical Center, Faculty of Medicine, University of Freiburg, Freiburg, Germany; 2 Department of Emergency Medicine, Cantonal Hospital of Neuchâtel, Neuchâtel, Switzerland

**Keywords:** Abroad elective, Medical elective, International elective, Travel Medicine, Global health, Internationalization, International health care, Medical education, Medical training

## Abstract

**Background:**

International medical electives are a well-established part of the curriculum of many western medical schools. It is widely accepted that these electives contribute to improved clinical examination and communication skills. Overseas electives also exert a strong influence over future career decisions and often pave the way for later international work. Whilst the positive outcomes are known, little information exists regarding elective preferences and destinations overall, information that could help optimise a safe learning experience and maximise the potential for one of the highlights of medical education. In order to obtain analytical data that could assist medical elective framework development, we systematically reviewed the two largest German online databases cataloguing abroad elective testimonies.

**Results:**

We identified 856 overseas elective reports uploaded within the last five years. European destinations were the most sought-after choice among German-speaking medical students. Interest in abroad electives in the United States (U.S.), a traditionally popular destination, was much lower than expected. U.S. elective reports accounted for only 3 % of long-term electives. Electives in low- and middle-income countries were generally less popular than electives in high-income countries. General surgery was the most popular elective discipline, followed by Emergency Medicine and Gynaecology and Obstetrics.

**Conclusions:**

We observed a large inhomogeneity in German-speaking medical students’ elective choices, potentially influenced by financial and organizational aspects as well as geopolitical developments. This highlights a crucial challenge for medical schools and other organizations involved in elective planning. In light of regional differences, our data suggest that a “one size fits all” preparation is not pertinent to optimize students’ elective experience. Country- or region-specific pre-departure trainings and more individualized elective frameworks might be necessary to address these differences and to ensure a safe learning experience for students.

**Supplementary Information:**

The online version contains supplementary material available at 10.1186/s12992-021-00742-z.

## Background

Medical electives are a popular and well-established part of the curriculum of many western medical schools [, 1, 2]. A result of the ease of travel and globalization of healthcare, an increasing number of medical students travel overseas for this important part of medical training, in a bid to develop medical competencies in new cultural settings [, , 3–5].

Perceived reported benefits include improved clinical examination skills, less dependency on technology (including expensive laboratory tests) as well as improved communication skills [, 4, 6, 7]. Destination specific, many students have reported an improved understanding of tropical diseases [7] and feeling better prepared to deal with underserved populations. Many students have also emphasized feeling more confident addressing immigrant and refugee health [8, 9], a (relatively) new feature of medical practice in the Western Hemisphere. Electives have also been shown to contribute to (medical) professional identity formation [10] and exert a strong influence over future career decisions [11], often paving the way for later international work. Traveling to low- and middle income countries may also be a life-changing experience, as students are confronted with different types of healthcare systems that are often less well equipped and frequently suffer from (strict) rationing policies.

Despite this myriad of benefits, very little is known about elective preferences overall, baring a few studies from the United Kingdom and Canada dating back to 2002 and 2008, respectively [4, 12]. Student choices regarding destination or preferred medical specialty as well as preparation material (including pre-departure courses and elective objectives) have not been extensively studied.

Moreover, it is uncertain which factors potentially contribute to the popularity of a specific destination. Yet, this information is important, as international medical electives have also been associated with health and safety risks [13, 14]. These include infectious disease, sexually transmitted infections as well as (trauma) injuries and mental health problems [11].

Reliable predictive data could help medical schools and other organisations involved in medical electives develop a framework to ensure a safe learning experience for their students. Adequate preparation and pre-departure training should be considered essential to maximise the learning experience, a feature which is especially true with the COVID-19 pandemic [15–17].

As travelling abroad for international electives is traditionally popular among German medical students [18, 19], we reviewed two large German databases for elective reports to identify patterns that may be helpful to help maximise a safe student experience, for what is generally recognised one the highlights of medical school [20].

### Medical education in Germany

In Germany, medical students are obliged to complete four one-month electives during the course of their studies; known as “Famulatur” [from the Latin “*famulus*”, which translates to “servant”] [21, 22]. These may be done nationally, though most students try to undertake at least one elective abroad [21, 22].

The last year of medical school is also comprised of three electives, though these last 3 months each, with mandatory rotations in Internal medicine and in General Surgery. This final year, known as “Praktisches Jahr” [German for “practical year”] represents another opportunity for students to travel overseas. In Germany, it is common for medical students to freely choose the discipline and destination of their third rotation. Upon completion of an elective, students often rate their experience and anonymously upload these reports (which include duration, country and home institution as well as the elective institution and elective discipline) to open-access databases. These databases can then be accessed by other German-speaking students (including those from Austria and Switzerland) planning their abroad electives.

In this article, we use the term “short-term elective” instead of “Famulatur-elective” and the term “long-term elective” instead of “PJ-elective”.

## Methods

In order to gain a better understanding of elective characteristics, we reviewed the two most popular German online databases cataloguing these elective testimonies. We focused on both short and long term electives, reviewing the “Famulatur-ranking” database (www.famulaturranking.de) as well as the PJ-Ranking database (www.pj-ranking.de). Both databases are mainly used by German, Swiss and Austrian students. For the purpose of this study, we defined an overseas elective as an elective performed outside of Germany, Switzerland and Austria.

In addition to a descriptive analysis, we also classified destinations according to their economic strength, based on World Bank Country and Lending Groups [23, 24]. Countries are divided among income groups according to their 2019 gross national income (GNI) per capita, calculated using the World Bank Atlas [23, 24]. With this approach, we aimed to investigate whether medical students preferred destinations in industrialized or developing countries.

### Data analysis

Reports from 2016 to 2020 were included, with analysis of the extrapolated data performed in January 2021 using GNU PSPP statistical software ([Version 0.8.5]. Free Software Foundation, Boston).

## Results

Within the last five years (2016–2020), we identified 856 overseas elective reports. 246 short-term (overseas) elective reports were uploaded to “Famulatur-ranking”; another 610 long-term (abroad) elective reports were uploaded to “PJ-ranking“.

On average, 55 short-term elective reports were uploaded per year between 2016 and 2019, whilst 145 long-term elective reports were uploaded yearly on average during the same timeframe. In both types of electives, numbers dropped considerably in 2020, with, respectively, 25 short-term elective reports and 31 long-term reports uploaded.

For the purpose of their electives overseas, German-speaking medical students travelled to 85 different countries worldwide. Tables 1 and 2 show the different elective destinations for short and long-term electives in detail.

**Table 1:** Short-term medical elective destinations of German-speaking medical students between 2016 and 2020: An overview.

Destination	2020	2019	2018	2017	2016	Total
Australia	0	2	2	0	0	4
Barbados	0	0	1	0	0	1
Belgium	1	1	3	2	1	8
Bolivia	0	1	0	0	0	1
Brazil	0	0	0	1	0	1
Cameroon	0	0	0	2	0	2
Canada	0	0	1	1	0	2
Chile	0	0	0	1	0	1
China	0	3	2	1	1	7
Colombia	1	0	0	0	1	2
Cyprus	0	0	1	0	0	1
Czech Republic	0	0	0	1	0	1
Denmark	0	3	1	1	0	5
Ecuador	0	0	0	2	0	2
El Salvador	0	0	0	0	1	1
Estonia	0	1	0	0	0	1
Finland	0	0	0	1	0	1
France	0	2	5	1	3	11
Gambia	0	1	1	0	0	2
Ghana	0	1	0	0	0	1
Greece	0	1	0	0	0	1
Guadeloupe	0	0	0	1	1	2
Hungary	0	0	0	2	0	2
India	1	0	2	1	3	7
Indonesia	0	0	1	0	0	1
Iraq	0	0	0	0	1	1
Ireland	0	4	1	1	1	7
Israel	0	0	0	0	4	4
Italy	13	7	6	8	9	43
Ivory Coast	0	1	0	0	0	1
Japan	1	2	1	3	4	11
Jersey	0	1	0	0	0	1
Luxembourg	1	1	0	0	1	3
Malta	0	2	0	0	0	2
Martinique	0	0	0	1	0	1
Mexico	2	2	0	0	0	4
Morocco	0	1	0	0	0	1
Namibia	0	2	0	0	0	2
Nepal	1	1	2	1	0	5
Netherlands	0	0	1	0	0	1
Nicaragua	0	0	0	1	0	1
Norway	0	0	0	0	1	1
Oman	1	0	0	0	1	2
Philippines	0	0	1	0	1	2
Portugal	1	0	0	0	0	1
Réunion	1	0	0	1	0	2
Russian Federation	0	0	0	0	1	1
Samoa	0	1	0	0	0	1
South Africa	1	2	2	0	3	8
South Korea	0	0	1	0	0	1
Spain	0	2	0	7	2	11
Sweden	0	2	2	1	4	9
Taiwan	0	1	0	0	0	1
Tanzania	0	3	2	3	2	10
Thailand	0	0	1	0	1	2
Trinidad and Tobago	0	0	0	1	0	1
Tunisia	0	0	0	1	0	1
Uganda	0	1	1	0	0	2
United Kingdom	0	2	7	9	7	25
United States of America	0	0	1	1	0	2
Vietnam	0	2	1	1	3	7
Total	**25**	**56**	**50**	**58**	**57**	**246**

**Table 2:** Long-term medical elective destinations of German-speaking medical students between 2016 and 2020: An overview.

Destination	2020	2019	2018	2017	2016	Total
Argentina	0	3	0	3	2	8
Australia	0	9	6	9	13	37
Bahamas	0	0	0	0	2	2
Belgium	2	0	1	1	2	6
Bolivia	0	0	0	0	1	1
Brazil	0	3	3	1	2	9
Canada	1	6	9	13	0	29
Chile	0	1	1	1	2	5
China	0	3	1	1	2	7
Colombia	0	4	3	5	3	15
Cuba	0	2	0	0	0	2
Denmark	0	1	5	0	2	8
Ecuador	0	0	0	2	0	2
Ethiopia	0	0	1	0	0	1
France	2	4	12	10	6	34
Ghana	1	3	1	5	1	11
Greece	0	0	0	2	0	2
Guadeloupe	0	0	1	0	1	2
Hong Kong	0	0	0	2	0	2
Hungary	0	1	0	0	0	1
India	2	2	2	0	0	6
Indonesia	0	1	1	0	0	2
Iran	0	0	2	0	0	2
Ireland	1	6	7	7	4	25
Israel	0	9	2	5	2	18
Italy	6	12	14	9	8	49
Japan	1	2	3	5	1	12
Kenya	1	2	1	4	0	8
Liechtenstein	0	1	0	0	0	1
Luxembourg	1	4	1	2	0	8
Malawi	0	0	0	0	1	1
Malaysia	0	0	0	1	1	2
Malta	1	0	1	0	1	3
Martinique	1	0	1	1	1	4
Mexico	0	4	3	0	0	7
Nepal	2	2	1	1	2	8
Netherlands	0	0	0	3	0	3
New Caledonia	0	0	2	0	0	2
New Zealand	2	4	2	4	0	12
Nicaragua	0	1	1	4	1	7
Norway	0	0	1	1	1	3
Palestinian Territories	0	1	1	0	0	2
Panama	0	0	1	1	0	2
Philippines	0	0	1	0	0	1
Poland	0	1	2	1	0	4
Portugal	0	2	1	1	0	4
Réunion	0	3	0	1	1	5
Rwanda	0	1	0	2	0	3
Samoa	0	1	0	1	0	2
South Africa	3	13	10	12	12	50
South Korea	0	0	1	3	0	4
Spain	0	5	11	6	2	24
Sri Lanka	1	0	6	2	3	12
Sweden	0	0	1	2	1	4
Taiwan	0	2	3	1	0	6
Tanzania	1	5	5	4	2	17
Thailand	0	0	2	0	0	2
Togo	0	1	0	0	0	1
Turkey	0	1	2	0	2	5
Uganda	1	0	0	0	0	1
United Arabian Emirates	0	1	0	0	0	1
United Kingdom	0	18	8	12	15	53
United States of America	1	5	7	2	4	19
Uruguay	0	3	0	3	0	6
Vietnam	0	1	2	2	3	8
Zambia	0	3	0	1	3	7
Total	**31**	**157**	**153**	**159**	**110**	**610**

**Fig. 1:**
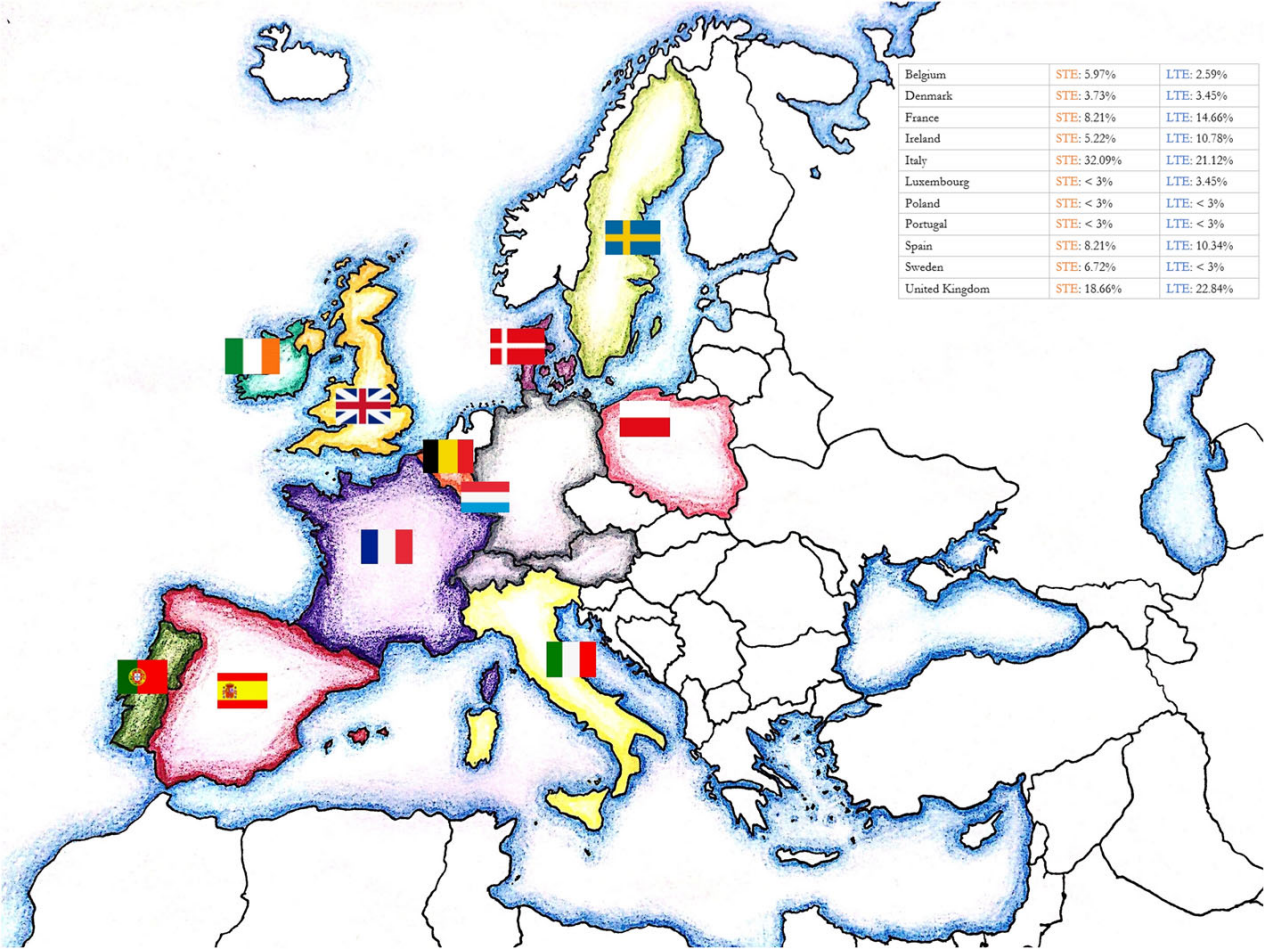
Popular European elective destinations of German-speaking medical students: an overview. Legend: STE = short-term elective, LTE = long-term elective

European destinations were the most popular choice for both short-term (almost 55 % of all reports (*n* = 134/246)) and long-term electives (approximately 38 % of all reports (*n* = 232/610)). Italy (32.09 % (*n* = 43/134)), the United Kingdom (18.66 % (n = 25/134)), Spain and France (each 8.21 % (*n* = 11/134)) were the most popular European destinations for short-term electives, whilst the United Kingdom (22.84 % (*n* = 53/232)) was the most frequently chosen European destination for long-term electives. Figure 1 shows the European destinations in detail. Noteworthy, Southern European countries attracted a large number of students, whereas Eastern European destinations were relatively unpopular. Southern European destinations accounted for approximately 50 % of European short-term elective reports (see Fig. 1).

Asian and African countries were also popular destinations for short-term electives. 18.70 % (*n* = 46/246) of students reported an elective in Asia, whilst approximately 13 % of students reported a short-term elective in Africa (*n* = 32/246). Japan was the most prominent Asian destination (23.91 %, (n = 11/46)), followed by China (15.22 % (*n* = 7/46)), India (15.22 % (*n* = 7/46)) and Nepal (10.87 % (*n* = 5/46)). Tanzania (31.25 % (*n* = 10/32)) and South Africa (25 % (*n* = 8/32)) were the most frequently chosen African countries.

Student’s interest in short-term electives in other geographical regions was less pronounced. Only 3.25 % (*n* = 8/246) of students reported an elective in North America, followed by South America, Central America and the Caribbean, and the Middle East (each 2.85 % (*n* = 7/246)). For short-term electives, Oceania attracted the fewest students (2.03 % (*n* = 5/246)).

For long-term electives, the situation appears to be different. Students favoured African countries (17.21 % (*n* = 105/610)) over Asian countries (12.62 % (*n* = 77/610)). South Africa (47.62 % (*n* = 50/105)) was by far the most popular destination, followed by Tanzania (16.19 % (*n* = 17/105)) and Ghana (10.48 % (*n* = 11/105)). With regard to Asia, Japan and Sri Lanka (15.58 % each (*n* = 12/77)) were the most frequently chosen destinations, followed by Vietnam and Nepal (10.39 % each (*n* = 8/77)). Approximately 9 % of students reported an elective in China (*n* = 7/77).

North America traditionally attracts many German-speaking students [21], and, unsurprisingly, about 9 % of students reported a long-term elective here (n = 55/610). Canada accounted for more than 50 % of these elective reports (*n* = 29/55), followed by the United States of America (34.55 % (*n* = 19/55)) and Mexico (12.73 % (*n* = 7/55)). With 8.69 % of elective reports, Oceania was also relatively popular for long-term electives (8.69 % (n = 53/610), with Australia attracting almost 70 % of these students (*n* = 37/53), followed by New Zealand (22.64 % (*n* = 12/53)).

Furthermore, South American countries were chosen more frequently (7.54 % (*n* = 46/610)) than countries in Central America and the Caribbean (3.11 % (n = 19/610)). Colombia (32.61 % (n = 15/46)), Brazil (19.57 % (n = 9/46)) and Argentina (17.39 % (*n* = 8/46)) were frequently visited for a medical elective. Among the Central American and Caribbean countries, Nicaragua was the most popular destination (36.84 % (n = 7/19)), followed by Martinique (21.05 % (n = 4/19)). Cuba, Panama, Guadeloupe and the Bahamas were equally popular (10.53 % (n = 2/19)).

Finally, 3.77 % of students reported a long-term elective in the Middle East (n = 23/610). Israel was by far the most popular destination (78.26 % (n = 18/23)), followed by Iran and the Palestinian Territories (each 8.70 % (n = 2/23)).

In addition to the most frequently chosen elective destinations, we also analysed the most popular elective disciplines (see Table 3). General surgery was the most popular discipline for both long-term (48.20 %, (n = 294/610)) and short-term (13.01 % (*n* = 32/246)) electives. Other popular disciplines for short-term electives included Anaesthesiology (10.98 % (n = 27/246)), Emergency Medicine (10.57 % (n = 26/246)), Gynaecology and Obstetrics (9.76 % (*n* = 24/246)), Internal Medicine (7.32 % (*n* = 18/246)) and Neurology (6.50 % (*n* = 16/246)).

**Table 3:** Short and long-term medical elective discipline preferences of German-speaking medical students between 2016 and 2020: An overview.

Discipline	Short-term elective	Long-term elective	Total
Anaesthesiology	27	17	44
Angiology	1	0	1
Cardiology	11	9	20
Cardiovascular Surgery	4	19	23
Dermatology	2	4	6
Emergency Medicine	26	10	36
Endocrinology	1	3	4
Forensic Medicine	0	1	1
Gastroenterology	3	7	10
General Medicine	3	0	3
General Surgery	32	294	326
Geriatrics	2	0	2
Gynaecology and Obstetrics	24	15	39
Haematology	2	6	8
Infectiology	5	3	8
Internal Medicine	18	98	116
Nephrology	1	5	6
Neurology	16	7	23
Neurosurgery	3	2	5
Ophthalmology	3	2	5
Oral and Maxillofacial Surgery	1	0	1
Orthopaedics	12	15	27
Otorhinolaryngology	6	1	7
Paediatric Surgery	2	5	7
Paediatrics	13	11	24
Plastic Surgery	7	19	26
Pneumology	1	2	3
Psychosomatic Medicine	1	0	1
Radiology and Neuroradiology	5	6	11
Rheumatology	0	2	2
Thoracic Surgery	0	2	2
Trauma Surgery	3	25	28
Tropical Medicine	4	0	4
Urology	5	5	10
Visceral Surgery	2	15	17
Total	**246**	**610**	**856**

**Fig. 2:**
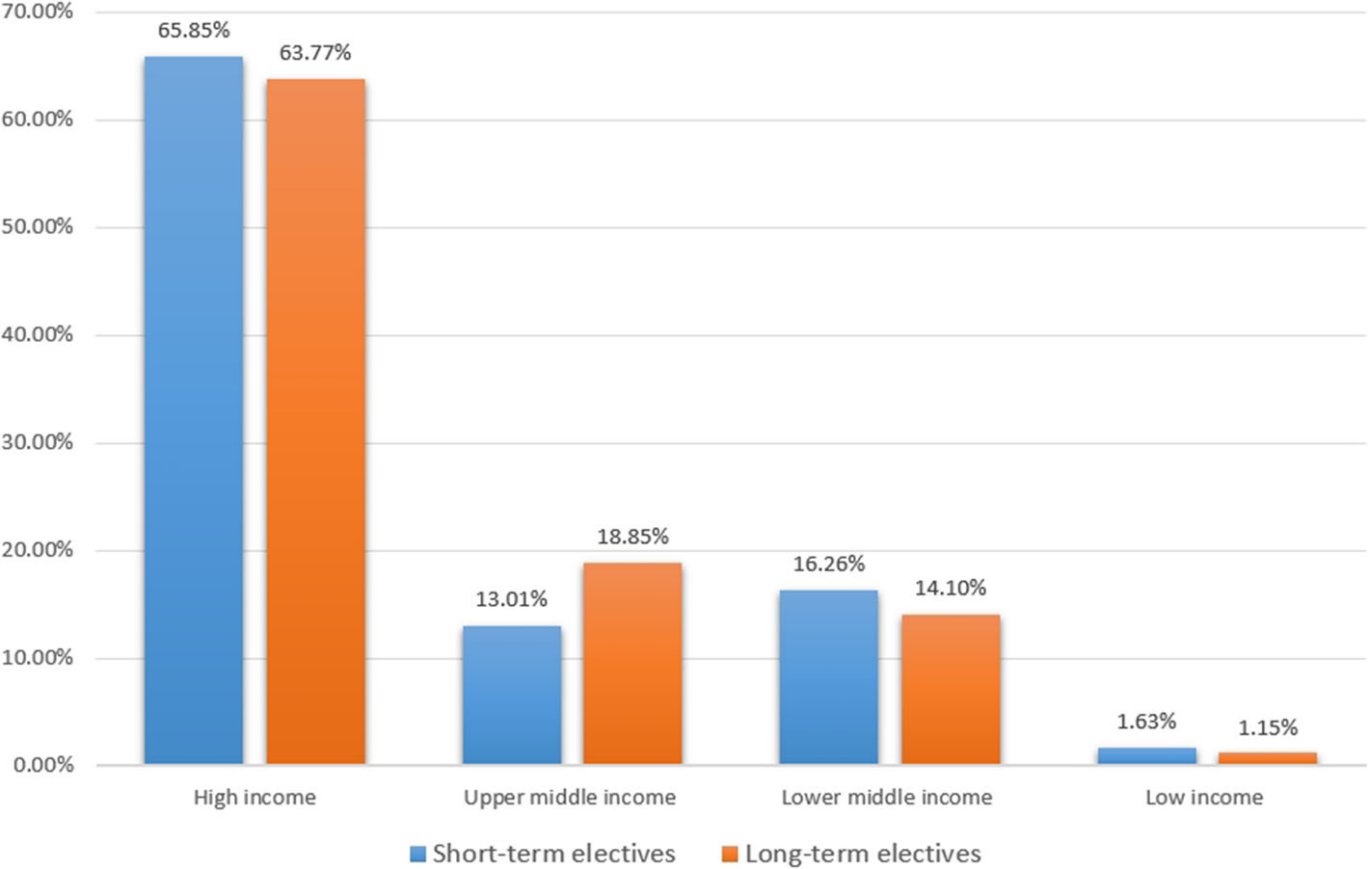
Elective destinations by income (according to the country classification table of the World Bank Country and Lending Groups): an overview

Electives in low- and middle-income countries were less popular than electives in high-income countries (Fig. 2). Almost 2/3 of students travelled to a high income country according to the country classification table of the World Bank Country and Lending Groups [20]. Low- and middle-income countries attracted approximately 1/3 of students. Some elective destinations are not listed in the World Bank Country and Lending Groups country classification table, thus “only” 835 (of 856) elective reports were submitted to this particular analysis.

The present analysis includes elective reports from a large number of universities throughout Germany. For a complete list, see Supplementary Information I.

## Discussion

The aim of this study was to gain a better understanding of popular abroad medical elective destinations and disciplines among German-speaking medical students. To the best of our knowledge, we are the first group to analyse German-speaking medical students’ elective preferences in such detail, and provide the largest review overall.

Reviewing two large German online databases, our study identified 856 abroad elective reports, which we submitted to a systematic analysis. Our study revealed that German-speaking medical students were more prone to spend their short- and long-term electives within Europe. Furthermore, we found that Asian and African countries attracted many students, as well. Surprisingly, the United States, a traditionally frequently selected destination among German students, accounted for only 3 % of long-term electives within the last five years.

Almost two decades ago, Miranda et al. surveyed medical students in the United Kingdom [25]. According to their study, 50 % of the survey participants went to another industrialized country and 40 % travelled to a developing country. Our results indicate similar findings, especially with regard to short-term electives. More than 50 % of medical students spent an abroad elective in an industrialized European country. For long-term electives, 38 % stayed within Europe. Electives in low- and middle-income countries accounted for approximately 1/3 of electives. In comparison to the British data from Miranda et al. [25], not much has changed within the last twenty years.

The ongoing ethical debate on disparities in international elective opportunities, which are skewed towards well-situated students from resource-rich nations [26, 27], has apparently no significant influence on German-speaking medical students’ choices. Electives in low- and middle-income countries are still popular and a significant proportion of students spend an elective in Africa.

Interpretation of our results in a larger context, however, warrants caution since many different external factors may have contributed to current trends among Germany-speaking medical students. Organizational and financial aspects as well as geopolitical factors may have influenced students in their elective choices.

### Financial and organizational aspects

It is conceivable that financial and organizational aspects play a pivotal role when it comes to the selection of a suitable (elective) destination. An international medical elective at a renowned institution in an Anglo-American country (especially in the United Kingdom, the United States or Australia) is probably still one of the strongest career boosts for German medical students. Such an elective may not only strengthen a student’s CV to enter more competitive residency programs but also demonstrates international experience as well as high proficiency in English [21]. The latter is of utmost importance when it comes to academic tasks and duties, including teaching activities, conference presentations as well as reading and publishing scientific literature.

However, securing a medical elective position in an English-speaking country and especially at an American institution has proven to be increasingly difficult [21], with a lengthy (often discouragingly) application process as well as high teaching and application fees [28, 29]. Although costs may vary from (elective) program to program, application fees can be as high as $1000 US Dollars per application [30]. Teaching fees usually depend on the elective length and can be as high as $6000 US Dollars for a 4 weeks elective [31]. Moreover, several universities in the United States nowadays only accept students in their last year of training, a fact that may also complicate the application process.

It is also conceivable that many German-speaking students do not have the financial capacity, or simply do not want to incur the costs, and therefore select destinations where institutions do not charge application and tuition fees for visiting students. In this context, it is also important to emphasize that the vast majority of German medical schools are state and tax-funded [19]. Therefore, tuition fees are significantly lower in Germany compared to the United States or the United Kingdom. Usually, only administrative fees are requested, ranging around 200–400 Euros per annum.

Destinations charging high tuition fees, such as universities in North America and Oceania, may thus only be available to more privileged students. Our data suggest that those who aim for an elective in an English-speaking country apparently prefer European destinations, (including the United Kingdom, Ireland and Malta) or African destinations (see Tables 1 and 2) with English as an official language, which cost less, and provide English-practice [21].

Another important factor for the popularity of European destinations may include the better funding opportunities. Specific examples include funding programs from the German Academic Exchange Service and the EU’s Erasmus + study exchange programme, which strengthens partnerships between European countries [32, 33]. These programs enable students to study abroad in another European country while being funded with up to 650 Euros per month. In comparison, scholarship programs for non-European countries are not as large as the EU’s Erasmus + study exchange programme and are often characterized by specific application requirements for medical students (e.g. special documents and certificates required) [34]. Thus, scholarships and funding programs that specifically promote an Inner-European exchange may also partially explain the popularity of European destinations in German-speaking medical students.

Given the high application fees in many countries, such as the United States and Canada, it is conceivable that students think twice before entering the intensive competition for an elective placement and before facing the risk of an application rejection (which goes along with a significant financial loss in case of non-refundable application fees).

### Geopolitical aspects

It is also interesting to examine our results from a geopolitical perspective. Regional instabilities and tensions in the Middle East are likely to daunt German medical students to spend an elective there. Ongoing military conflicts and civil war in Libya, Syria, and Yemen as well as the weakening role of the United States as a power balancer in the Middle East resulted in deepened ﻿tensions and regional competition [35]. The fact that German medical students are advised to undertake electives in safe locations with comparable (hospital) infrastructures may therefore explain the low interest in electives in this particular region.

On the other hand, as a result of the increasing number of students going abroad, the competition for popular elective placements in the United States is growing [36]. Within the last decades, populous countries like China and India experienced a continuous process of internationalization and globalization [37]. For example, Chinese students now make up the largest group of international students in the United States and have even surpassed those from India [37, 38]. A recent paper analysing the internationalization of medical education in China revealed that North America (41.53 %) is one the top choices among Chinese medical students [39]. It is not inconceivable that these relatively new and strong competitors may change the field of international electives. In fact, a rapidly increasing international competition for a limited number of elective spots could hypothetically lead European students to stay in Europe for their electives. As many European institutions and universities require foreign students to provide language certificates for the respective national language, competition may not be as pronounced as it is the case with English-speaking countries, e.g. the United States or Canada.

Geopolitical developments during the COVID-19 pandemic may have also accounted for the decline in total abroad electives in 2020. Many countries took uncompromising measures in response to the COVID-19 pandemic [40] and imposed (international) travel bans as well as reinforced border controls and national lockdowns. These measures are likely to have contributed to the reduced number of international electives, as many universities and institutions rapidly closed their visiting programs for foreign (international) students [41].

Another factor that warrants mentioning is the recent changes in immigration policies in many countries, foremost the United States of America [42]. Tightened immigration laws and stiffened visa regulations (as observed in the United States during the last years [42, 43]) may had a direct impact on students’ ability to enter the country.

### General Implications

There are no internationally mandated standards for international medical elective program coordinators. Therefore, Watson and colleagues recently proposed a series of generalizable recommendations that may help support elective coordinators in promoting successful elective experiences for students [44]. Recommendations cover 11 different themes, including general policies and elective responsibilities, travel advisories and occupational risk assessment, pre-departure training programs and post-return debriefings, funding and finances, clinical routine, accommodation and safety, ethical behaviour and social accountability, and finally, health and wellbeing.

Although these recommendations are of paramount importance, our results also reveal an enormous challenge for those who try to put such recommendations into practice: the large variety and inhomogeneity of elective destinations. Given the large number of potential destinations in considerably varying environments, it becomes obvious that a general guideline can only serve as a rough directive. Our results highlight the need for more destination-specific guidelines and directives. We hope that our data supports medical schools and organisations involved in medical elective planning to develop a framework that ensures a safe learning experience for their students. Now that the most popular destination of German-speaking students are known, specific guiding principles and policies could be developed that further enhance student safety while being abroad.

### Strengths and Limitations

Our analysis has several weaknesses and strengths that are worth mentioning. We present a large dataset including more than 800 elective reports covering almost all medical faculties across Germany.

To the best of our knowledge, a comparably detailed analysis over such a large timeframe has not been conducted before. Our data could be of utmost importance for those involved in the development of abroad elective frameworks and guidelines. The fact that we also revealed the most popular elective disciplines may support universities and other institutions to tailor subject-specific recommendations and guidelines.

Of note, the present analysis also has several limitations that warrant further discussion. The first challenge corresponds to the sample size, which in itself is a reflection of the non-mandatory aspect of elective report uploading. Although we investigated a relatively large timeframe (5 years), we identified less than 1000 reports in total - a pale figure when considering that the total number of medical students enrolled in Germany alone is around 80,000 students. The possibility that only highly motivated students upload elective reports may potentially lead to selection bias and we do not know whether our results are applicable for all German-speaking medical students.

In addition to that, no personal interviews were carried out - our analysis is based solely on written reports from two open-access databases. Therefore, reasoning behind students’ elective choices can only be inferred. Direct elective testimonies based on qualitative (semi-structured) interviews would have enriched the present analysis. Unfortunately, both interrogated databases do not routinely save student contact data; thus, such personal interviews are beyond the scope of this paper.

Ultimately, it also difficult to verify the authenticity and reliability of the data. Hypothetically, one could upload fake (elective) reports, yet we believe this to be unlikely in light of the efforts required to upload an elective testimony (uploading is a multi-step process requiring plenty of data and time).

Nonetheless, overall, we believe that our data represents a good cross-sectional image of German-speaking medical students.

## Conclusions

The presented results give insight into the most popular elective destinations and disciplines of German-speaking medical students. We provide data that may support medical schools and other organizing institutions to develop country-specific pre-departure trainings with the aim to improve the safety and health of medical students going abroad for international electives. We also suggested various factors that may influence students’ elective choices. These include both financial and organizational aspects as well as geopolitical developments. Those factors warrant further investigation and need to be confirmed in future studies.

## Supplementary Information

**Additional file 1:**

## Data Availability

All data associated with this paper will be made available upon reasonable request.
