# Supplementary material for: German-speaking medical students on international electives: an analysis of popular elective destinations and disciplines
Source: Global Health. 2021 Aug 16;17:90. doi: 10.1186/s12992-021-00742-z (PMC8365125; doi:10.1186/s12992-021-00742-z)
Supplement: Supplementary file 1 — Additional file 1: [file 12992_2021_742_MOESM1_ESM.docx]

The present analysis includes electives reports from a large number of Universities throughout Germany, including Aachen University, Humboldt University of Berlin, Ruhr University Bochum, Friedrich-Wilhelms University of Bonn, Dresden University, Heinrich-Heine University Düsseldorf, Friedrich-Alexander University Erlangen-Nürnberg, University of Duisburg-Essen, Goethe University Frankfurt am Main, Albert-Ludwig University of Freiburg, Justus Liebig University Gießen, Georg-August University of Göttingen, Ernst-Moritz-Arndt University of Greifswald, Martin-Luther University of Halle-Wittenberg, Hamburg University, Hannover University, Ruprecht-Karls University of Heidelberg, Saarland University, Friedrich-Schiller University of Jena, Christian-Albrechts University of Kiel, Köln University, Leipzig University, Lübeck University, Otto-von-Guericke University Magdeburg, Johannes-Gutenberg University Mainz, Marburg University, Ludwig-Maximilian University of Munich, Technical University of Munich, Münster University, Regensburg University, Rostock University, Eberhard-Karl University of Tübingen, University of Ulm and University of Würzburg.
